# Changes in mortality and years of life lost due to lung cancer in Poland, 2000–2016

**DOI:** 10.1186/s12967-020-02354-4

**Published:** 2020-05-06

**Authors:** Małgorzata Pikala, Monika Burzyńska, Irena Maniecka-Bryła

**Affiliations:** Department of Epidemiology and Biostatistics, The Chair of Social and Preventive Medicine of the Medical University of Lodz, Żeligowskiego 7/9, Łódź, 90-742 Poland

**Keywords:** Lung cancer, Neoplasms, Years of life lost, Mortality, Epidemiology, Trends, Poland

## Abstract

**Background:**

The aim of the study was to evaluate trends of mortality and the number of years of life lost due to lung cancer in Poland, in the period 2000–2016.

**Methods:**

The study material was 375,151 death certificates of all inhabitants of Poland who died in the period 2000–2016 due to lung cancer. In order to calculate the number of years of life lost, the authors used indices: SEYLL_p_ (Standard Expected Years of Life Lost per living person), SEYLL_d_ (per deaths), APC (Annual Percentage Change) and AAPC (Average Annual Percentage Change).

**Results:**

The standardized death rate (SDR) due to lung cancer decreased in the analyzed period from 74.5 to 68.3 per 100,000 population (AAPC = −0.6%). The most rapid decrease was noted in the years 2008–2011 (APC = –2.2%). With regards to males, SDR decreased from 148.8 to 114.5 (AAPC = –1.7%), whereas in females, it increased from 25.7 to 37.6 (AAPC = 2.3%). The SEYLL_p_ index, calculated per 100,000 inhabitants, increased from 1189.9 in the year 2000 to 1250.5 in the year 2016. The trend and pace of changes fluctuated. In 2000–2008, the SEYLL_p_ index was increasing at a pace of 0.7%. This growth was followed by a decrease at a pace of −1.2%, noted in 2008–2011. After the year 2011, the indices started to grow at an annual pace of 0.4%. AAPC in the whole study period was 0.3%. Increased mortality in females was responsible for the increase in the number of lost years of life. SEYLL_p_ values in this sex group increased from 464.8 in the year 2000 to 774.7 in the year 2016 (APC = 3.3%).With regards to males, SEYLL_p_ values, calculated for 100,000 male population, decreased in the analyzed period from 1961.1 to 1758.3.

**Conclusions:**

Lung cancer still poses a serious epidemiological problem in Poland and the number of years of life lost due to this cause reflects social and economic implications of premature lung cancer-related mortality. There is a great need to educate, particularly women, and show effective ways of quitting smoking.

## Background

An increased incidence of neoplastic diseases is observed all around the world. This growing trend is contributed by ageing, affecting populations as well as exposure to carcinogenic factors. According to the International Agency for Research on Cancer (IARC) 18.1 million new cases of neoplastic diseases were detected in 2018. Besides 9.6 million people died due to them. 23.4% of cases of neoplasms are noted in Europe, which is also characterized with the highest percentage of neoplastic deaths (20.3%) [1]. In Poland, neoplastic diseases are the second cause of mortality, following cardiovascular diseases, being the greatest contributors of mortality [2]. In 2016, neoplasms contributed to 27% of all deaths [3].

Lung cancer is nowadays the most often diagnosed neoplasm. Besides, it is also the leading cause of cancer deaths worldwide [4]. In 2018, it made up 11.6% of all diagnosed neoplastic diseases and 18.4% of the total number of neoplasm-related deaths [5]. Poland is characterized by very high mortality due to lung cancer. In 2016, the standardized death rate by this cause was the second largest among all countries of the European Union (Table 1).

**Table 1:** Daily tobacco smoking rate among people aged ≥ 15 years and age-standardized death rates due to lung cancer in European Union countries in 2016

	Daily tobacco smoking rate among people aged ≥ 15 years			Age-standardized death rates due to lung cancer		
	Both sexes	Male	Female	Both sexes	Male	Female
Austria	22.9	23.5	22.3	46.6	64.6	33.0
Belgium	22.7	25.3	20.3	57.8	91.2	32.6
Bulgaria	28.0	35.5	21.0	43.8	79.3	16.5
Croatia	31.9	35.0	29.1	66.1	116.2	30.4
Cyprus	28.4	41.4	15.0	37.5	61.6	16.6
Czechia	25.6	29.9	21.5	51.8	82.1	29.6
Denmark	15.0	14.6	15.3	66.8	76.0	60.2
Estonia	24.0	32.0	17.1	51.0	99.8	23.7
Finland	15.7	17.6	13.8	39.4	60.6	24.2
France	27.3	29.8	24.9	48.7	78.0	25.5
Germany	24.2	27.8	20.7	50.6	73.0	33.6
Greece	34.3	44.7	24.6	60.9	107.1	23.0
Hungary	25.1	29.4	21.2	90.1	137.9	58.2
Ireland	18.8	20.1	17.6	57.1	68.0	48.3
Italy	19.6	23.3	16.2	48.7	80.2	25.1
Latvia	30.6	44.6	19.1	46.5	103.6	15.2
Lithuania	22.0	31.8	13.9	44.3	96.8	14.1
Luxembourg	18.0	19.7	16.3	51.9	80.9	28.5
Malta	20.0	24.5	15.5	42.5	70.1	21.8
Netherlands	20.8	21.9	19.8	66.0	88.2	50.3
Poland	23.4	28.2	19.0	68.9	114.6	38.1
Portugal	18.0	25.0	11.8	37.1	66.4	14.9
Romania	24.6	31.8	18.0	54.0	95.8	21.8
Slovakia	22.5	29.2	16.3	51.3	92.8	24.9
Slovenia	19.2	21.4	17.0	58.3	91.7	33.8
Spain	24.7	26.7	22.9	48.4	86.5	18.0
Sweden	10.8	9.9	11.6	37.6	41.4	35.1
United Kingdom	17.5	19.6	15.4	59.3	71.9	49.7

*Source* WHO global report on trends in prevalence of tobacco smoking 2000–2025, second edition [11], Eurostat statistics [34]

Carcinogens which contribute to the development of lung cancer include physical, chemical and genetic factors as well as low physical activity, overweight and obesity [6, 7]. However, exposure to cancerogenic agents, found in nicotine smoke is the greatest risk factor, responsible for 30% of all cancer deaths [8, 9]. Scientific research reveals that 85–90% of all histological types of lung cancer are related to active and passive nicotine smoking. The incidence is directly proportional to the smoking period and the number of smoked cigarettes. Smoking 20 cigarettes per day causes a 25-fold increase in the risk of lung cancer, whereas smoking 40 cigarettes per day contributes to even a 60-fold increase in the risk of developing this type of cancer [10]. According to “World Health Organization (WHO) global report on trends in prevalence of tobacco smoking”, in the year 2016, 23.4% of the Polish population, aged 15 and above, were daily nicotine smokers [11]. The mean percentage of daily smokers in 28 countries of the European Union was 21%. It was the highest in Greece (34.3%), Croatia (31.9) and Latvia (30.6%) and the lowest in Finland (15.7), Denmark (15.0%) and Sweden (10.8%) (Table 1). Males, rather than females, were daily smokers. In Poland, 28.2% of males and 19.0% of females are daily smokers.

Lung cancer management has been modified over the last 20 years. However, the disease is still associated with a very poor prognosis. An analysis of 5-year survival, conducted as part of the CONCORDE-3 study, revealed that in most of sixty-six analyzed countries, including Poland, the 5-year survival rate diagnosed between 2010 and 2014, ranged from 10 to 19%. The highest rate was noted in Japan (33%). In comparison with patients, diagnosed in the period 1995–1999, the 5-year survival rate for 36 countries, increased by 5–10%, whereas in China, Japan and Korea, it increased by more than 10% [12]. 5-year survival rates in Poland, in the periods 2000–2002 and 2003–2005, increased both in males and females, from 10.8% to 11.9% and from 15.7% to 16.9%, respectively, and their values were similar to mean European rates [13]. Despite relatively positive trends in the survival of lung cancer, the disease is still one of disorders with the worst prognosis.

Treatment of neoplasms is a serious economic burden for any society. The diseases also generate a great loss of income, resulting from work absenteeism or premature death. In 2009, researchers from Oxford University and King’s College in London made an economic analysis of expenses incurred on medication and medical care as well as a loss of earnings caused by the disease or care provided for the sick. All 27 countries of the European Union were included in the analysis. It revealed that the annual cost of management of neoplastic diseases in the European Union is 126 billion Euros. The highest costs are generated by lung cancer, which is responsible for 15% of expenses incurred on neoplasms in Europe (18.8 billion Euros). This disease affects younger people. Thus, premature mortality, decreasing productivity, is the main contributor.

According to estimates, annual costs associated with treatment of malignant neoplasms are 3.6 billion Euros. Of this number, 1.3 billion Euros are costs related to decreased productivity, being a direct result of premature cancer-related mortality. With regards to management of all neoplastic diseases, lung cancer generates the highest costs, i.e. 716 million Euros, which is equal to 20% of the total costs related to treatment of neoplasms in Poland [14].

The aim of the study was to evaluate trends of mortality and the number of years of life lost due to lung cancer in Poland, in the period 2000–2016.

## Methods

The study material was a database including 6384,495 death certificates of all inhabitants of Poland who died in the period 2000–2016. Of this number, 375,151 people died of lung cancer (according to the International Statistical Classification of Diseases and Health Related Problems—Tenth Revision—ICD-10, coded as C33-34). The data were provided by the Department of Information of the Polish Central Statistical Office. The procedure of coding causes of death in Poland is performed in a similar way to the one carried out in the majority of countries in the world, by basing on the so called primary cause of death, or the disease which triggered a pathological process, leading to death.

The authors calculated crude deaths rates (CDR) and standardized death rates (SDR).

$$CDR = \frac{k}{p}*100,000$$

Where k—number of lung cancer deaths; p—number of people.

The standardization procedure was performed with the use of direct method, in compliance with the European Standard Population, updated in 2012 [15].

$$SDR = \frac{{\sum\limits_{i = 1}^{N} {\frac{ki}{pi}wi} }}{{\sum\limits_{i = 1}^{N} {wi} }}$$

Where k_i_ is the number of lung cancer deaths in this i-age group, p_i_ is the population size of this i-age group, w_i_ is the weight assigned to this i-age group, resulting from the distribution of the standard population, N—stands for the number of the age groups.

Years of life lost were calculated and analyzed by the method described by Christopher Murray and Alan Lopez in Global Burden of Disease (GBD) [16]. The SEYLL index (Standard Expected Years of Life Lost) is used to calculate the number of years of life lost by the studied population in comparison to the number of years lost by the referential (standard) population.

There are some methods of calculating lost years of life and the main difference between them is a point of reference, i.e. the level of mortality which is considered “ideal”. In the GBD 2010 study, WHO experts recommend using life tables based on the lowest noted death rate for each age group in countries with population above 5 million [17].

In this study, the SEYLL index was calculated according to the following formula:

$$\varvec{SEYLL} = \mathop \sum \limits_{{\varvec{x} = 0}}^{\varvec{l}} \varvec{d}_{\varvec{x}} \varvec{e}_{\varvec{x}}^{\varvec{*}}$$

Where ${\text{e}}_{\text{x}}^{ *}$—life expectancy, based on GBD 2010 life tables, d_x_—number of deaths at age x, x—age at which the person died, l—last age which the population reaches.

The authors also applied the SEYLL per person (SEYLL_p_) index, which is a ratio of SEYLL and the size of population, calculated per 100,000 inhabitants, and the SEYLL per death (SEYLL_d_) index, being a ratio of SEYLL and the number of deaths due to a particular cause i.e. it expresses the number of YLL calculated per one dead person [18].

The analysis of time trends has been carried out with the use of joinpoint models and Joinpoint Regression program, a statistical software package developed by the U.S. National Cancer Institute for the Surveillance, Epidemiology and End Results Program [19]. This method is an advanced version of linear regression, where the time trend is expressed with a broken line, being a sequence of segments joined in joinpoints. In these points, the change of the value is statistically significant (p < 0.05). We have also calculated the annual percentage change (APC) for each segment of broken lines and the average annual percentage change (AAPC) for the whole study period with corresponding 95% confidence intervals (CI).

## Results

In the years 2000–2016, 375,151 people died of lung cancer in Poland. More than 20,000 people die of this disease each year. In the analyzed period, the number of deaths due to this cause was increasing: in the year 2000, it was 20,002, whereas in 2016, the number of deaths was 23,833 (Table 2). In 2000 and in 2016, crude death rates (CDRs), calculated for 100,000 population were: 52.3 and 62.0, respectively. AAPC for this period was 1.0% but we observed that within this 17-year analyzed period, the trend changed twice. In the years 2000–2008, APC was 1.3%; in the years 2008–2011, CDRs decreased at an annual pace of −0.7%, whereas in the years 2011–2016, they started to grow again, at a pace of 1.5% (Table 5).

**Table 2:** Number of deaths and values of CDR, SDR, SEYLL, SEYLL_p_ and SEYLL_d_ due to lung cancer in Poland in 2000–2016

Year	Number of deaths	CDR (per 100,000)	SDR (per 100,000)	SEYLL	SEYLL_p_ (per 100,000)	SEYLL_d_ (per deaths)
2000	20,002	52.3	74.5	455,166	1189.9	22.8
2001	20,627	53.9	75.7	466,483	1219.8	22.6
2002	21,254	55.6	76.9	476,405	1246.5	22.4
2003	21,035	55.1	75.0	468,797	1227.5	22.3
2004	21,206	55.6	74.3	471,503	1235.1	22.2
2005	21,515	56.4	74.5	475,165	1245.3	22.1
2006	21,775	57.1	74.2	477,765	1253.1	21.9
2007	22,148	58.1	74.2	483,184	1267.7	21.8
2008	22,523	59.1	74.4	486,948	1276.9	21.6
2009	22,348	58.6	72.4	480,881	1259.9	21.5
2010	22,374	58.1	71.3	478,417	1241.7	21.4
2011	22,251	57.7	69.4	473,611	1228.9	21.3
2012	22,650	58.8	69.4	479,057	1243.2	21.2
2013	22,655	58.9	68.1	475,294	1234.7	21.0
2014	23,210	60.3	68.8	478,850	1244.5	20.6
2015	23,745	61.8	69.2	486,013	1264.4	20.5
2016	23,833	62.0	68.9	480,614	1250.5	20.2

The value of CDRs as well as direction and pace of changes hugely differed for males and females. In the male group, the rates were stable. In the year 2000 CDR calculated for 100,000 males was 86.2, whereas in the year 2016 it was 87.1 (AAPC = 0.0) (Tables 3 and 5). In the group of females, we observed a rapid increase in CDRs values, i.e. from 20.4 in 2000 to 38.5 in 2016 (APC = 4.0) (Tables 4 and 5).

**Table 3:** Number of deaths and values of CDR, SDR, SEYLL, SEYLL_p_ and SEYLL_d_ due to lung cancer among men in Poland in 2000–2016

Year	Number of deaths	CDR (per 100,000)	SDR (per 100,000)	SEYLL	SEYLL_p_ (per 100,000)	SEYLL_d_ (per deaths)
2000	15,984	86.2	148.8	363,528	1961.1	22.7
2001	16,397	88.5	150.0	372,392	2010.2	22.7
2002	16,725	90.4	151.7	374,560	2023.9	22.4
2003	16,335	88.4	146.0	364,086	1969.5	22.3
2004	16,565	89.7	145.2	368,071	1992.8	22.2
2005	16,562	89.7	144.0	364,508	1975.2	22.0
2006	16,658	90.4	141.9	364,804	1979.8	21.9
2007	16,582	90.1	139.6	360,195	1956.4	21.7
2008	16,880	91.7	140.2	363,385	1973.3	21.5
2009	16,392	88.9	133.5	350,592	1902.4	21.4
2010	16,204	86.9	129.6	344,750	1848.2	21.3
2011	15,988	85.7	124.7	338,622	1815.2	21.2
2012	16,206	86.9	123.8	341,830	1832.9	21.1
2013	16,002	85.9	119.9	333,526	1790.3	20.8
2014	15,847	85.1	117.2	325,298	1747.1	20.5
2015	16,261	87.4	118.0	331,718	1783.6	20.4
2016	16,190	87.1	114.6	326,916	1758.3	20.2

**Table 4:** Number of deaths and values of CDR, SDR, SEYLL, SEYLL_p_ and SEYLL_d_ due to lung cancer among women in Poland in 2000–2016

Year	Number of deaths	CDR (per 100,000)	SDR (per 100,000)	SEYLL	SEYLL_p_ (per 100,000)	SEYLL_d_ (per deaths)
2000	4018	20.4	25.7	91,638	464.8	22.8
2001	4230	21.5	26.8	94,091	477.2	22.2
2002	4529	23.0	28.0	101,845	516.7	22.5
2003	4700	23.9	28.7	104,711	531.4	22.3
2004	4641	23.6	27.7	103,432	524.9	22.3
2005	4953	25.1	29.2	110,657	561.6	22.3
2006	5117	26.0	29.8	112,961	573.4	22.1
2007	5566	28.2	31.6	122,989	624.2	22.1
2008	5643	28.6	31.6	123,562	626.6	21.9
2009	5956	30.2	32.7	130,289	660.1	21.9
2010	6170	31.0	33.4	133,667	672.5	21.7
2011	6263	31.5	33.2	134,989	678.9	21.6
2012	6444	32.4	33.7	137,226	690.1	21.3
2013	6653	33.5	34.1	141,769	713.6	21.3
2014	7363	37.1	37.3	153,552	773.2	20.9
2015	7484	37.7	37.2	154,295	777.7	20.6
2016	7643	38.5	38.1	153,698	774.7	20.1

**Table 5:** Time trends of CDR, SDR, SEYLL_p_ and SEYLL_d_ due to lung cancer in Poland in 2000–2016—joinpoint regression analysis

	Number of joinpoints	Years	APC (95% CI)	AAPC (95% CI)
Total				
CDR	2	2000–2008	1.3* (1.0; 1.7)	1.0* (0.4; 1.6)
		2008-–2011	−0.7 (−3.8; 2.5)	
		2011–2016	1.5* (0.8; 2.2)	
SDR	2	2000–2008	−0.2 (−0.6; 0.1)	−0.6* (−1.2; 0.0)
		2008–2011	−2.2 (−5.5; 1.2)	
		2011–2016	−0.3 (−1.0; 0.5)	
SEYLL_p_	2	2000–2008	0.7* (0.4; 1.0)	0.3 (−0.3; 0.8)
		2008–2011	−1.2 (−4.1; 1.8)	
		2011–2016	0.4 (−0.2; 1.1)	
SEYLL_d_	1	2000–2012	−0.6* (−0.6;−0.6)	−0.7* (−0.8; −0.7)
		2012–2016	−1.1* (−1.4; −0.9)	
Men				
CDR	2	2000–2008	0.5* (0.1; 0.9)	0.0 (-0.7; 0.6)
		2008–2011	−2.1 (−5.8; 1.7)	
		2011–2016	0.3 (−0.5; 1.2)	
SDR	2	2000–2008	−1.0* (−1.4; −0.6)	−1.7* (−2.4; −1.0)
		2008–2011	−3.6 (−7.3; 0.2)	
		2011–2016	−1.7* (−2.5; −0.8)	
SEYLL_p_	1	2000–2006	−0.1 (−0.9; 0.7)	−0.9* (−1.2; −0.5)
		2006–2011	−1.3* (−1.7; −1.0)	
SEYLL_d_	1	2000–2012	−0.7* (−0.7; −0.6)	−0.7* (−0.8; -0.7)
		2012–2016	–1.0* (–1.3;–0.8)	
Women				
CDR	0	2000–2016	4.0* (3.7; 4.2)	
SDR	0	2000–2016	2.3* (2.1; 2.6)	
SEYLL_p_	0	2000––2016	3.3* (3.1; 3.6)	
SEYLL_d_	1	2000–2012	−0.4* (−0.6; −0.3)	–0.7* (−0.9; –0.6)
		2012–2016	–2.0* (–3.0;–1.0)	

*p < 0.05

Some of the observed changes possibly resulted from the age of the population. Thus, in order to eliminate the impact of this factor, the authors calculated standardized death rates (SDRs). In 2000, SDR was 74.5 per 100,000 population. In 2016, its value decreased to 68.9 (AAPC = −0.6%). A decline trend was observed in the whole analyzed period and the decrease was most rapid in the years 2008–2011, i.e. −2.2%.

**Fig. 1:**
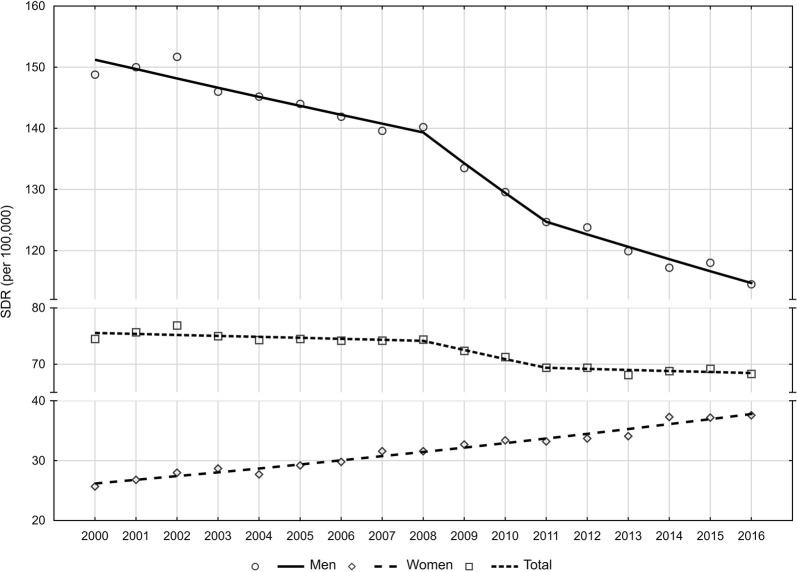
Time trends of standardized death rates (SDR) due to lung cancer in 2000–2016 in Poland

Similarly to CDR, also the trend of SDR changes was different for males and females (Fig. 1). In the male group, SDRs decreased from 148.8 in 2000 to 114.6 in 2016 (AAPC = −1.7%) (Tables 3 and 5), whereas in females, SDRs increased from 25.7 in 2000 to 38.1 in 2016 (AAPC = 2.3%) (Tables 4 and 5). Disproportions regarding lung cancer-related mortality, observed in the two sexes, are gradually decreasing. In the year 2000, SDR in males was 5.8 higher than in females but in 2016, the rate was 3.0 times higher.

**Fig. 2:**
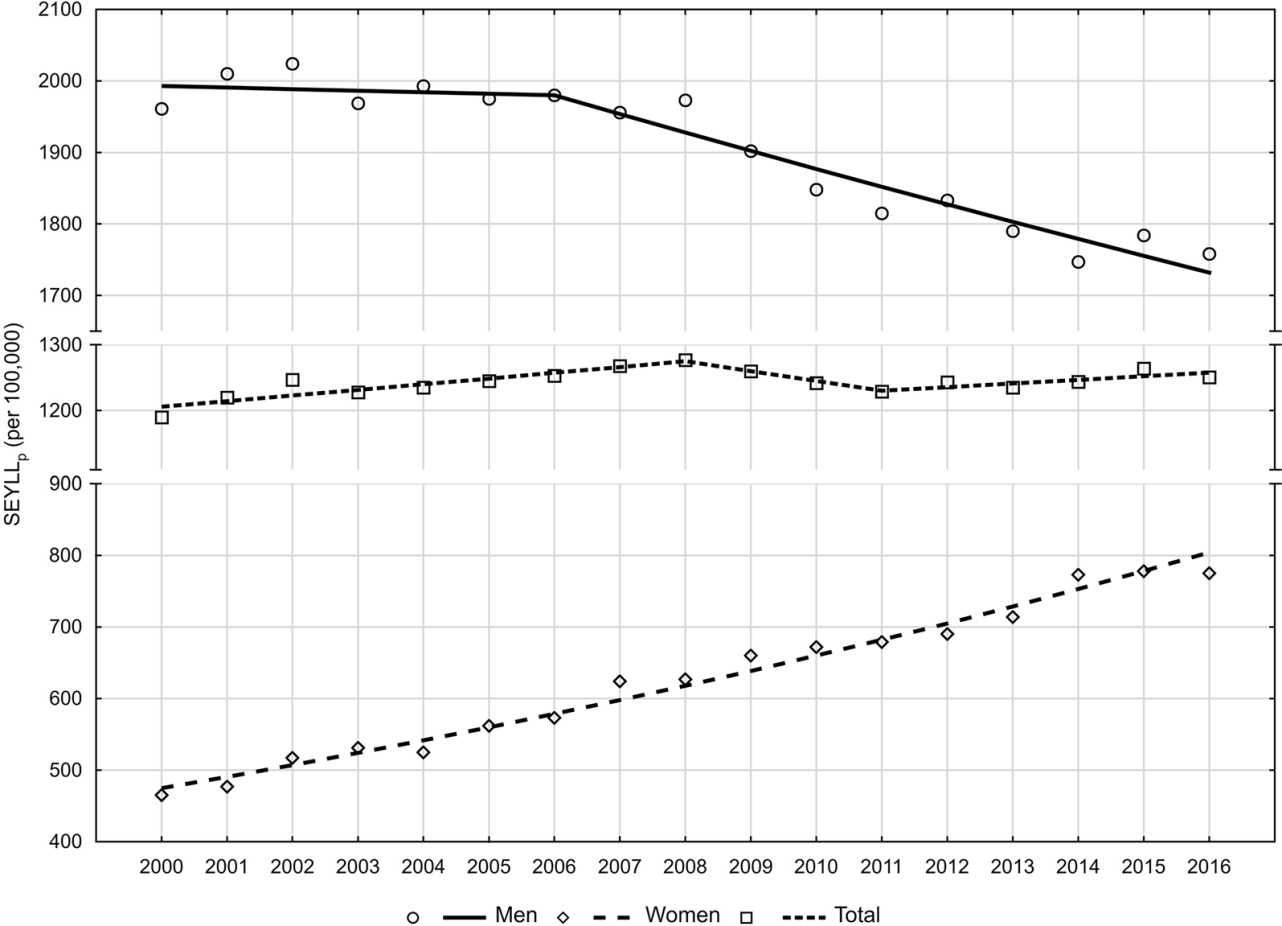
Time trends of standard expected years of life lost per living person (SEYLL_p_) in 2000–2016 in Poland

Premature mortality due to lung cancer contributes to a loss of years of life in the Polish population. In the year 2000, the number of standard expected years of life lost (SEYLL) was 455,166. In 2016, the SEYLL increased to 480,614 (Table 2). The SEYLL_p_ index, calculated for 100,000 population, increased from 1189.9 in 2000 to 1250.5 in 2016. The direction and pace of changes fluctuated (Fig. 2). After a growth at a pace of 0.7%, noted in the years 2000–2008, between the years 2008 and 2011 the SEYLL_p_ index started to decrease at a pace of −1.2%. After the year 2011, we observed an increase at an annual pace of 0.4%. The AAPC value in the whole analyzed period was positive, i.e. 0.3% (Table 5).

An increase in the number of years of life lost was mostly contributed by increased mortality in the female group. In males, SEYLL_p_ index values were decreasing in the whole study period. In the years 2000–2006, the decline was slight (APC = −0.1%); after 2006, the decline pace increased up to –1.3%. As a result, SEYLL_p_ index values decreased from 1961.1 per 100,000 males in 2000 to 1758.3 in 2016 (Tables 3 and 5). In the female group, SEYLL_p_ index values were growing at the same annual pace of 3.3%. This contributed to an increase in the SEYLL_p_ index from 464.8 in 2000 to 774.7 in 2016 (Tables 4 and 5).

**Fig. 3:**
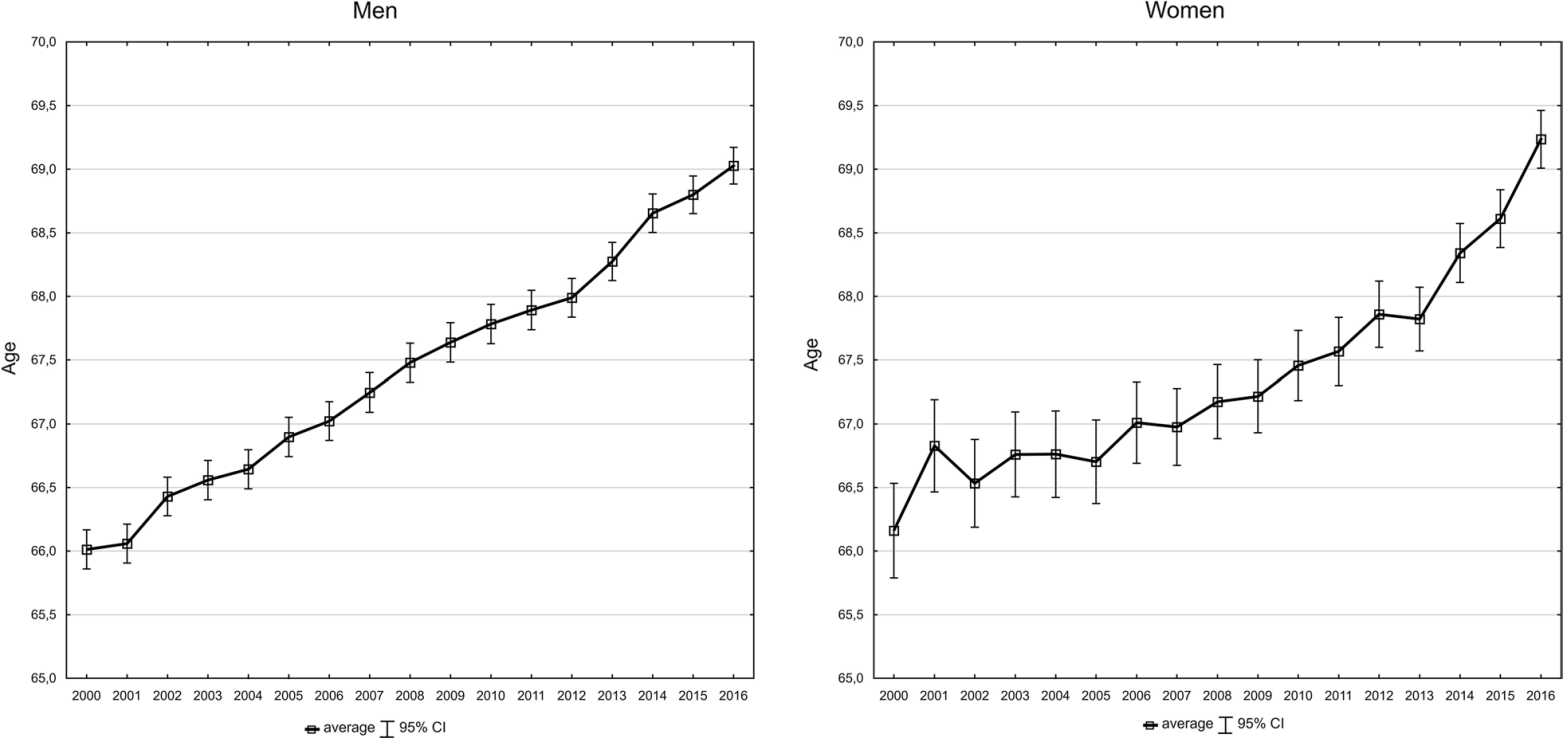
The average age of men and women who died of lung cancer in 2000–2016 in Poland

**Fig. 4:**
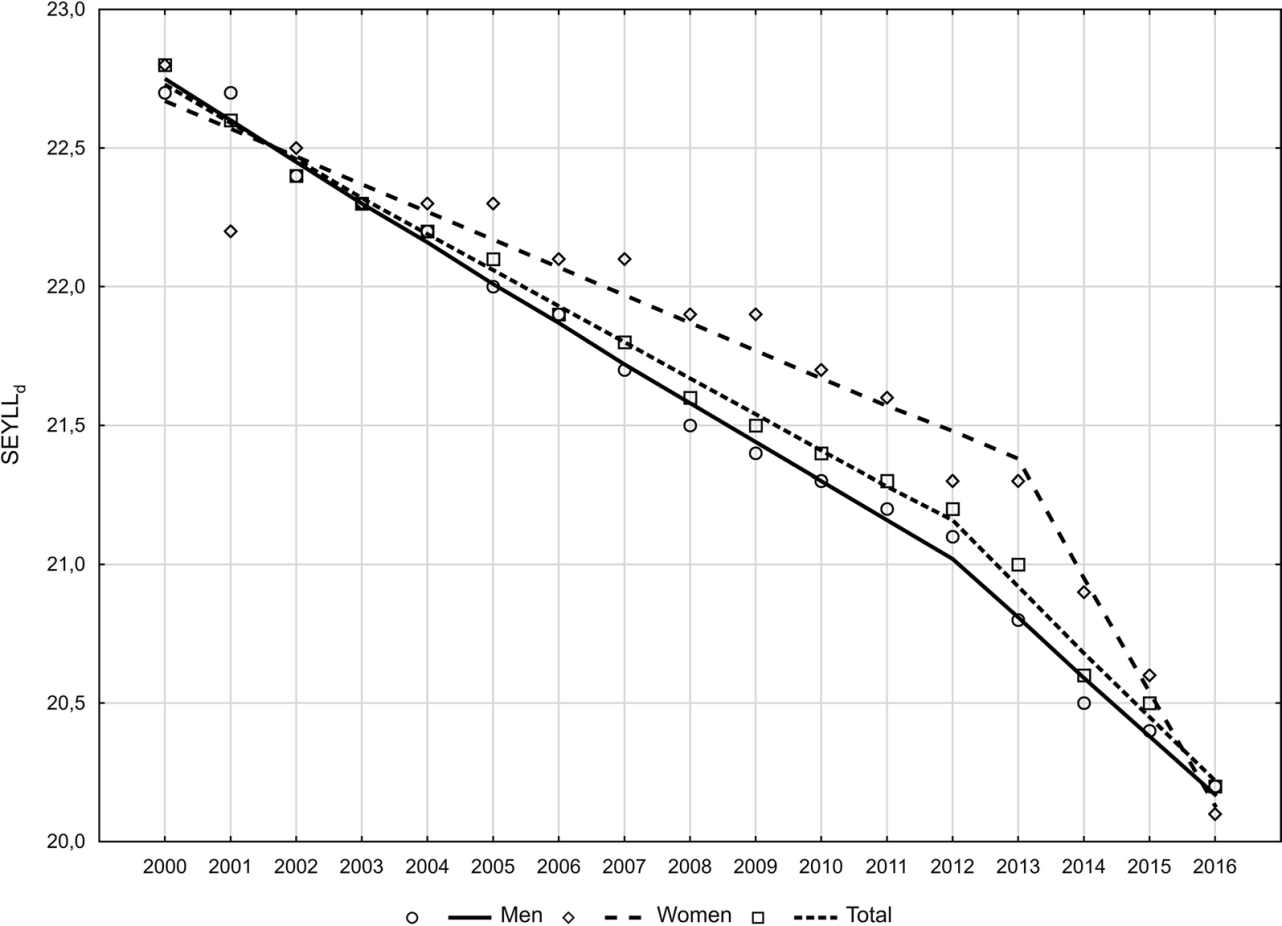
Time trends of standard expected years of life lost per deaths (SEYLL_d_) in 2000–2016 in Poland

The average age of death from lung cancer increased in 2000–2016 from 66.0 to 69.0 in the group of men and from 66.2 to 69.2 in the group of women (Fig. 3). We noted related to this fact a constant decrease in the number of years of life lost, calculated for one person who died due to lung cancer (SEYLL_d_), and the decline pace increased from the year 2012 (Fig. 4). Each person who died in the year 2000 due to this cause lost on average 22.8 years of life. In the years 2000–2012, APC was –0.6%, whereas in the years 2012–2016, it was –1.1%. In consequence, in 2016, the SEYLL_d_ index was 20.2 (Tables 2 and 5). Decreased SEYLL_d_ index values were observed both in males and females. In males, it declined from 22.7 in 2000 to 20.2 in 2016. APC was −0.7% in the years 2000–2012 and −1.0% in the years 2012–2016 (Tables 3 and 5). With regards to females, the SEYLL_d_ index calculated for the year 2000 was 22.8. After a decline in the years 2000–2012, at an annual pace of −0.4% and a more rapid decrease, i.e. –2.0% in the years 2012–2016, the SEYLL_d_ index was 20.1 years (Tables 4 and 5).

## Discussion

Lung cancer is the most common neoplasm, affecting males in Poland. Its incidence has been decreasing for almost two decades. However, we observed that the number of females who died due to this neoplasm was gradually increasing in the years 2000–2016. As a result of this increase, mortality trends due to lung cancer and breast cancer, intersected in 2003, which implies that lung cancer is the most common mortality contributor in females, too. Similar tendencies were earlier noted in other European countries, e.g. Denmark, Holland, Sweden, Great Britain and Ireland [20]. Positive changes in mortality trends in males and negative in females can be largely contributed by a changing percentage of tobacco smokers. The largest percentage of smokers in Poland was observed in 1982 and it amounted to almost 70% among men and almost 50% among women [21]. After 1982, in the group of men, the percentage of smokers was steadily decreasing, amounting to 40.9% in 1996, 34.1% in 2004, 31.0% in 2009 and 28.0% in 2014 [22, 23]. When analyzing changes in the frequency of smoking, it should be noted that the positive effects of quitting smoking in the form of reducing the number of deaths, occur after a certain delay—after about 3 decades [24]. The rapid decline in the number of smokers in 1982–1996 (by almost 30 percentage points) may be the reason for the increase in the rate of decline in life years lost due to lung cancer after 2006 observed in our study. In the group of women, the percentage of smokers is decreasing at a very slow pace: 19.4% in 1996, 19.4% in 2004, 18.0% in 2009 and 17.2% in 2014 [22, 23]. The highest levels of smoking were observed in the generation of women born between 1940 and 1960. The observed cohort effect and the slow rate of decline in the percentage of smoking women mean that the incidence and the resulting mortality are still showing an upward trend that will persist for some time [25]. This serious problem was also confirmed by the authors of this study who showed that in the years 2000–2016, the group of females demonstrated a significant increase in the number of years of life lost due to lung cancer. This was reflected by SEYLL_p_ values which were growing at an annual pace of 3.3%.

Differences in mortality trends and the number of lost years of life due to lung cancer in males and females were also observed in Germany, where in the years 1952–2012, the number of Years Potential Life Lost (YPLL) increased from 6.6 to 11.3 in females. With regards to males, this value increased in the period 1978–1989 but started to decline gradually. Finally, in the years 2006–2012, this value got stable [26]. Similar differences in trends of incidence and lung cancer-related mortality trends in males and females were also noted in the United States, where in the study period (1975–2015), indices in males became the highest possible in the year 1988. In the female group, they were increasing until the year 2006 [27].

With regards to lung cancer, we can modify risk factors, which may substantially lessen the threat of this disease. In Poland, Tobacco Attributable Fraction is estimated to be about 80–90% in males and about 60–70% in females [21], whereas in Serbia it is 82.8% in males and as much as 90.2% in females [28]. In non-smoking Poles, the incidence of pulmonary malignancies is very low, i.e. less than 5 cases of lung neoplastic diseases are diagnosed in 100,000 population [29]. Research and comparative analyses of the epidemiological situation in Poland and other countries show that elimination of tobacco appears to be effective in combating lung cancer. It is worth comparing time trends of mortality, caused by lung malignancies in young males in Poland and Hungary. In the 1960s’ and 1970s’ the time trends were identical. After implementation of an anti-tobacco policy in Poland, in the 1990s’, the trends were no longer the same. In Hungary, the incidence of lung malignancies became one of the highest in the world, whereas in Poland, the incidence started to decline. In Hungarian females, mortality rates appeared to be almost three times higher than in Poland [30]. Undoubtedly, decreased exposure to nicotine results in a decreased risk of lung cancer [31]. Implementation of multidirectional program aiming at reduction of tobacco smoking-related health consequences in Poland resulted in a decreased consumption of tobacco, which in turn, significantly reduced the incidence of lung cancer and lung cancer-related mortality in tobacco smokers [32].

## Limitations

Quality of the analyses performed on the mortality statistics depend on the completeness and accuracy of the information contained in the death certificate and the proper and precise description of the cause of death. Poland is a country with 100% completeness of death registration. In order to standardize death causes, which are subject to further statistical analyses, it was determined that the doctor who pronounces death is responsible for filling in the death card, into which he or she puts the primary, secondary and direct death cause, whereas qualified teams of doctors are responsible for coding death causes according to the ICD-10 classification. The data relating to 2012 shows that the cause of more than 28% of deaths (about 109.000) were inaccurately described, however, in the majority of cases (78.500) it concerned deaths due to cardiovascular diseases. Significantly fewer incorrect codes number concerns other causes of death, including cancer [33].

## Conclusions

Lung cancer still poses a serious epidemiological problem and the number of years of life lost due to this cause reflects social and economic implications of premature lung cancer-related mortality. While in the group of men lung cancer mortality and the number of years of life lost due to this cause systematically decreases, in the group of women a steady upward trend of both indices has been observed in Poland since 2000. Therefore, there is a great need to intensify educational and preventive programs, particularly for women. There is also a need to further monitor changes in this area.

## Data Availability

The datasets used and/or analysed during the current study are available from the corresponding author on reasonable request.

## Abbreviations

AAPC: Average annual percentage change
APC: Annual percentage change
CDR: Crude deaths rate
CI: Confidence interval
GBD: Global Burden of Disease
IARC: The International Agency for Research on Cancer
ICD-10: The International Statistical Classification of Diseases and Health Related Problems—Tenth Revision
SDR: Standardised death rate
SEYLL: Standard expected years of life lost
SEYLL_d_: Standard expected years of life lost per deaths
SEYLL_p_: Standard expected years of life lost per living person
WHO: World Health Organization
YPLL: Years potential life lost
